# Association Analysis of the Tryptophan Hydroxylase 2 Gene Polymorphisms in Patients with Methamphetamine Dependence/Psychosis

**DOI:** 10.2174/157015911795017335

**Published:** 2011-03

**Authors:** Hideaki Kobayashi, Hiroshi Ujike, Nakao Iwata, Toshiya Inada, Mitsuhiko Yamada, Yoshimoto Sekine, Naohisa Uchimura, Masaomi Iyo, Norio Ozaki, Masanari Itokawa, Ichiro Sora

**Affiliations:** 1 Department of Biological Psychiatry, Tohoku University Graduate School of Medicine, Sendai 980-8574, Japan; 2 Department of Neuropsychiatry, Okayama University Graduate School of Medicine, Dentistry and Pharmaceutical Sciences, Okayama 700-8558, Japan; 3 Department of Psychiatry, Fujita Health University School of Medicine, Aichi 470-1192, Japan; 4 Department of Psychiatry, Seiwa Hospital, Institute of Neuropsychiatry, Tokyo 162-0851, Japan; 5 Department of Psychogeriatrics, National Institute of Mental Health, National Center of Neurology and Psychiatry, Tokyo 187-8553, Japan; 6 Division of Medical Treatment & Rehabilitation, Center for Forensic Mental Health, Chiba University, Chiba 260-8670, Japan; 7 Department of Neuropsychiatry, Kurume University School of Medicine, Kurume 830-0011, Japan; 8 Department of Psychiatry, Graduate School of Medicine, Chiba University, Chiba 260-8670, Japan; 9 Department of Psychiatry, Nagoya University Graduate School of Medicine, Nagoya 466-8550, Japan; 10 Schizophrenia Research Project, Tokyo Institute of Psychiatry, Tokyo 156-8585, Japan; 11 Japanese Genetics Initiative for Drug Abuse (JGIDA), Japan

**Keywords:** Single nucleotide polymorphism, SNP, variation, serotonin, human, Japanese, MAP, abuse.

## Abstract

There is a growing evidence that serotoninergic systems modulate dopaminergic neurotransmission. We analyzed the association between the variations in the brain tryptophan hydroxylase 2 (*TPH2*) gene, a rate limiting enzyme for serotonin biosynthesis, and methamphetamine (METH) dependence/psychosis in a Japanese population. We found ten single nucleotide polymorphisms (SNPs) and two polynucleotide polymorphisms in *TPH2* gene exons and exon-intron boundaries. A total of 162 patients and 243 controls were used for the association analysis between these polymorphisms and METH dependence/psychosis. No significant differences were observed in either genotypic or allelic frequencies between METH dependent/psychotic patients and controls. A global test of differentiation among samples based on haplotype frequencies showed no significant association. With respect to latency of psychosis, prognosis of psychosis, and spontaneous relapse, we found no significant association with these SNPs. These results suggest that the *TPH2* gene variants may not be a factor in vulnerability to METH dependence/psychosis.

## INTRODUCTION

Methamphetamine (METH) is a psychomotor stimulant with high liability for abuse, and METH abuse has become a very serious social problem in Japan [1]. Chronic METH abusers have been shown to have persistent dopaminergic deficits [2, 3]. In animals, amphetamine elevates extracellular dopamine levels in the mesolimbic circuits [4, 5]. There is growing evidence that serotonergic systems modulate dopaminergic neurotransmission. For example, the mesocorticolimbic dopamine system is under inhibitory control by the serotonin system, which exerts its actions *via* serotonin receptor subtypes [6, 7].

Acute and chronic administration of METH markedly decreases the activity of tryptophan hydroxylase (TPH) [8, 9], the rate-limiting enzyme in the biosynthesis of serotonin [10]. TPH2 (or neuronal TPH) was identified as a second isoform of TPH in 2003 [11, 12]. In contrast to TPH1, which is expressed predominantly in the pineal gland and the periphery, TPH2 mRNA is expressed in the raphe nuclei [11]. Since the identification of TPH2, there have been numerous association analyses between *TPH2* gene variants and psychiatric diseases. For example, associations have been observed between *TPH2* variants and bipolar disorder [13-18], suicidal behavior in major depression [19-21], the response to selective serotonin reuptake inhibitors (fluoxetine and/or citalopram) [22, 23] and emotional regulation in healthy subjects [24-28]. These reports indicate that polymorphic variants in the *TPH2* gene may have a role in the pathophysiology of a wide range of psychiatric disorders and emotional regulation. A recent study of heroin addiction also showed an association with *TPH2* variants in Hispanics and African-Americans [29].

The purpose of this study was (1) to identify novel sequence variations in all coding exons as well as exon-intron boundaries of the *TPH2* gene in Japanese, and (2) to investigate whether these polymorphisms and/or haplotypes were associated with METH dependence/psychosis.

## MATERIALS AND METHODS

### Subjects

One-hundred sixty-two unrelated patients with METH dependence/psychosis (130 males and 32 females; mean age 37.4±12.0 years) meeting ICD-10-DCR criteria (F15.2 and F15.5) were used as case subjects; they were outpatients or inpatients of psychiatric hospitals. The 243 control subjects (168 males and 75 females; mean age 35.4±11.5 years) were mostly medical staff members who had neither personal nor familial history of drug dependence or psychotic disorders, as verified by a clinical interview. All subjects were Japanese, born and living in the northern Kyushu, Setouchi, Chukyo, Tokai, and Kanto regions. This study was approved by the ethical committees of each institute of the Japanese Genetics Initiative for Drug Abuse (JGIDA), and all subjects provided written informed consent for the use of their DNA samples for this research [30]. After informed consent was obtained, blood samples were drawn and genomic DNA was extracted by the phenol/chloroform method. 

### Defining Variants of the *TPH2* Gene

Initially, 16 METH dependent/psychotic patient samples were used to identify nucleotide variants within the *TPH2* gene (GenBank accession no. AC090109). Exons 1 to 11 and exon-intron boundaries were amplified by polymerase chain reaction (PCR) using a thermal cycler (Astec, Fukuoka, Japan), and the products were sequenced in both directions using BigDye terminators (Applied Biosystems, Foster City, CA) by an ABI Genetic analyzer 3100 (Applied Biosystems).

Genotyping of each polymorphism except in exon 11 was performed by PCR amplification using the relevant primers listed in Table **1** followed by sequencing using the same primers in both directions. Genotyping of polymorphisms in exon 11 was performed by PCR amplification using 9F and 11R primers followed by sequencing using 10F, 11F, and 11R primers.

### Patient Subgroups

For the clinical category analysis, the patients were divided into two subgroups by three different clinical features. (A) Latency of psychosis from first METH intake: less than 3 years or more than 3 years. The course of METH psychosis varied among patients, with some patients showing psychosis sooner after the first METH intake, as previously reported [30, 31]. Because the median latency was three years, this time point was used as the cutoff in defining the two groups. (B) Duration of psychosis after the last METH intake: transient (<1 month) or prolonged ( ≧1 month). Some patients showed continuous psychotic symptoms even after METH discontinuation, as previously reported [30, 31]. Patients with the transient type showed a reduction of psychotic symptoms within one month after the discontinuation of METH consumption and the beginning of treatment with neuroleptics. Patients with the prolonged type showed a psychotic symptoms continued for more than one month even after the discontinuation of METH consumption and the beginning of neuroleptic treatment. (C) Spontaneous relapse: present or not. It has been well documented that once METH psychosis has developed, patients in the remission phase are liable to spontaneous relapse without reconsumption [30, 31].

### Statistical Analysis

The Hardy-Weinberg equilibrium of genotypic frequencies in each SNP was tested by the chi-square test. The level of statistical significance was set at α= 0.05. The allelic and genotypic frequencies of patients and control groups were compared using the chi-square test. Locus by locus linkage disequilibrium (LD) was evaluated by D’ and r^2^, which were calculated by the haplotype frequencies using the appropriate formula in the Excel program. A global test of differentiation among samples based on haplotype frequencies was performed using the Arlequin program available from http://anthropologie.unige.ch/arlequin [32].

## RESULTS

**Fig. (1):**
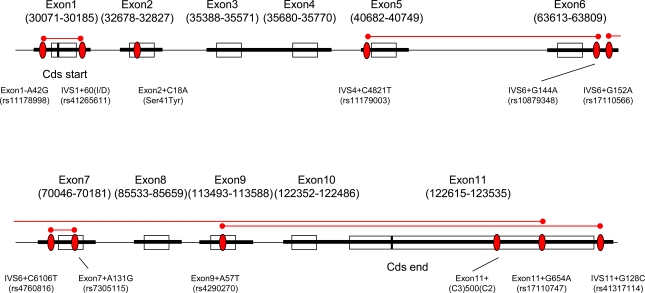
Location and linkage disequilibrium mapping of the *TPH2* gene polymorphisms. All the coding exons and their regions were taken from the NCBI database under accession number AC090109. Red ovals indicate the polymorphic positions, solid black lines the analyzed regions, and solid red lines the LD block.

To identify polymorphisms in the *TPH2* gene, all coding exons (1 to 11) and exon-intron boundaries were analyzed using genomic DNA from 16 Japanese METH-dependent/psychotic subjects. Ten single nucleotide polymorphisms (SNPs) and two insertion / deletion polymorphisms were identified. One polymorphism, Exon11+(C3) 500(C2), was novel (Table **2**). Two SNPs, rs7305115 (Exon7+A131G) and rs4290270 (Exon9+A57T), were synonymous mutations and Eon2+C18A was a non-synonymous mutation. Three linkage disequilibrium (LD) regions were found, rs11178998 (Exon1-A42G) to rs41265611 (IVS1+60(I/D)), rs11179003 (IVS4+C4821T) to rs10879348 (IVS6+G144A), and rs4760816 (IVS6+C6106T) to rs7305115 (Exon7+A131G), in the sense that all genotypic patterns in all 16 samples analyzed were the same. Each one of the SNPs was chosen and a total of nine SNPs were genotyped for further analysis. LD mapping was analyzed by using SNPs having minor allele frequencies of over 10% in both samples (Table **4**). LD was observed from rs17110566 (IVS6+G152A) to rs17110747 (Exon11+G654A) and from rs4290270 (Exon9+A57T) to rs41317114 (IVS11+G128C) (Fig. **1** and Table **3**). 

Association analyses were performed on these nine polymorphic positions using 162 METH dependent/psychotic patients and 243 controls. Genotypic frequencies in these SNPs were within the Hardy-Weinberg expectations. No significant differences were found in the allelic or genotypic frequencies of these SNPs between the METH dependent/psychotic patients and the controls (Table **4**). Since the minor allele frequency of the Exon11+(C3)500(C2) SNP was less than 1% in controls, this SNP was excluded from the haplotype analysis. No significant difference (P=0.448) was observed in a differentiation test between all pairs of samples based on haplotype frequencies by the Arlequin program.

Subcategory analyses were conducted on the clinical parameters (latency of psychosis, prognosis of psychosis, and spontaneous relapse). SNPs having minor allele frequencies of over 10% in both samples were used for this analysis: rs17110566 (IVS6+G152A), rs4760816 (IVS6+C6106T), rs4290270 (Exon9+A57T), rs17110747 (Exon11+G654A), and IVS11+G129C. No significant associations with clinical parameters were observed (Table **5**).

## DISCUSSION

We analyzed the *TPH2* gene polymorphisms in a Japanese population and found ten SNPs and two insertion/deletion variants, among which one variant was novel. However, we failed to identify any variants or haplotypes in the *TPH2* gene examined in this study which were associated with METH dependence/psychosis.

Exon2+C18A is a nonsynonymous SNP and the corresponding amino acid is changed from Ser to Tyr at peptide position 41 (S41Y). This SNP was reported as C2755A by Lin and colleagues in a Han Chinese population [14]. They transfected plasmids containing full-lengthTPH2 protein–encoding sequences with two alternative alleles into SH-SY5Y cells and found that the amount of serotonin in SH-SY5Y cells expressing the 41Y allele was about 36% lower than in cells expressing the 41S allele. Despite the strong scientific rationale for studying polymorphisms in the *TPH2* gene in METH dependence/psychosis, we could not identify any variants or haplotypes associated with the phenotype. These results were comparable to those for cocaine use. Both cocaine and METH increase extracellular dopamine in the brain, and increased dopamine in the nucleus accumbens is thought to underlie the reinforcing effects of drugs of abuse [5, 33]. The association of cocaine dependence in subjects of African descent with TPH2 SNPs was analyzed by Dahl and colleagues, but they failed to identify any SNPs that were associated with the cocaine-dependent phenotype [34]. The disparity between these results and the previously reported results for heroin addiction [29] suggest that the *TPH2* gene has little effect in psychostimulants with the characteristics of indirect dopaminergic agonists.

Our results indicate that the *TPH2* gene variations may not be vulnerability factors in METH dependence/psychosis, and indeed that they are likely to make a small or no contribution to the development of METH dependence/psychosis.

## Figures and Tables

**Table 1:** Primers Used in this Study

Exon	Forward		Reverse	
Exon 1	1F	CCT TAT GTA TTG TTC TCC ACC ACC	1R	GTT GAG CAC GCA GTG ATT GGC ACA
Exon 2	2F	CCA CTA GAT GAT GTC TTA GAC CAT	2R	CTG ACC TCC TAA CCT GGC AAT AGT
Exon 3,4	3F	GTA CTT GGC ACC TTG CTT AAG ATG	3R	TGG AAG TCT GCT CTC AGT TAT GGG
Exon 5	4F	GCT CAA CTA AGC CAT TCT GCT TAC	4R	GTA GCA CTT GGC ATG TGG CTC ACA
Exon 6	5F	GAT CCT TTC AGA CGC TCA TGT GCT	5R	CAT ACT CAT GTA GCC CAG CAC AGC
Exon 7	6F	GTG CGG TAA GCA TCA CTT TCG ATT	6R	CAG ATG AGG AGT CTG ATC CTT CAG
Exon 8	7F	GAA GTC CCA GCA TTG ATG AAC TGT	7R	GGC TAA GCT GAG TAA TTC TGA CAG
Exon 9	8F	CAG GAA GCG TAA GAC TCT TAG TAG	8R	GTC AGT AGG ATC ACT GCT AGC TCA
Exon 10, 11	9F	CCT GCA CAC AGG AGA GTT CCA TAT	9R	CAT GCT GGC AAC AAC ATA GTT CCA
	10F	CAA TCC CTA CAC ACA GAG TAT TGA	10R	CAT TCC AAC TGC TGT GTT ACC TCA
	11F	GAT CTA AGC CTT TCC TCT GTG TTC	11R	GAC ACA GAA ACA CAT GCA AGC ACT

**Table 2:** *TPH2* Gene Variants Found in the Japanese Population

Position1)	Location	rs Number2)	SNP Name	Variation	Function
30029	5' side	rs11178998	Exon1-A42G	A/G	
30241	Intron 1	rs41265611	IVS1+60(I/D)	TCT/del	
32694	Exon 2		Exon2+C18A3)	C/A	nonsynonymous (Ser41Tyr)
40601	Intron 4	rs11179003	IVS4+C4821T	C/T	
63953	Intron 6	rs10879348	IVS6+G144A	G/A	
63961	Intron 6	rs17110566	IVS6+G152A	G/A	
69915	Intron 6	rs4760816	IVS6+C6106T	C/T	
70176	Exon 7	rs7305115	Exon7+A131G	A/G	synonymous (Pro312Pro)
113549	Exon 9	rs4290270	Exon9+A57T	A/T	synonymous (Ala375Ala)
123114	Exon 11		Exon11+(C3)500(C2)	C3/C2	
123268	Exon 11	rs17110747	Exon11+G654A	G/A	
123663	3' side	rs41317114	IVS11+G128C	G/C	

1) Position: nucleotide position number in the NCBI nucleotide database under accession number AC090109.

2) rs number: NCBI SNP database.

3) This SNP was reported as C2755A [14].

**Table 3:** Linkage Disequilibrium Mapping of the *TPH2* Gene

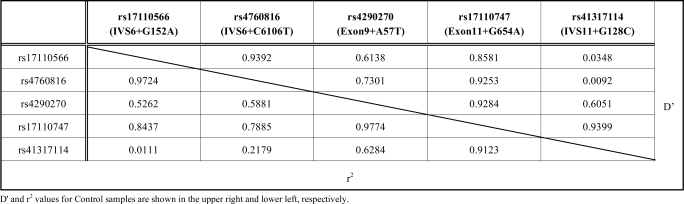

D' and r^2^ values for Control samples are shown in the upper right and lower left, respectively.

**Table 4:** Genotypic and Allelic Distribution of the *TPH2* gene SNPs in the METH Dependent/Psychotic Patients and the Control Groups

SNP	Group	Genotype (%)			P	Allele (%)		P
rs11178998 (Exon1-A42G)		A/A	A/G	G/G		A	G	
	METH	130 (80%)	29 (18%)	3 (2%)	0.102	289 (89%)	35 (11%)	0.617
	Control	197 (81%)	46 (19%)	0 (0%)		440 (91%)	46 (9%)	
Exon2+C18A		C/C	C/A	A/A		C	A	
	METH	146 (90%)	16 (10%)	0 (0%)	0.914	308 (95%)	16 (5%)	0.807
	Control	222 (91%)	21 (9%)	0 (0%)		465 (96%)	21 (4%)	
rs10879348 (IVS6+G144A)		G/G	G/A	A/A		G	A	
	METH	136 (84%)	26 (16%)	0 (0%)	0.975	298 (92%)	26 (8%)	0.920
	Control	206 (85%)	37 (15%)	0 (0%)		449 (92%)	37 (8%)	
rs17110566 (IVS6+G152A)		G/G	G/A	A/A		G	A	
	METH	123 (76%)	35 (22%)	4 (2%)	0.552	281 (87%)	43 (13%)	0.406
	Control	173 (71%)	64 (26%)	6 (2%)		410 (84%)	76 (16%)	
rs4760816 (IVS6+C6106T)		C/C	C/T	T/T		C	T	
	METH	28 (17%)	85 (52%)	49 (30%)	0.314	141 (44%)	183 (56%)	0.200
	Control	57 (23%)	121 (50%)	65 (27%)		235 (48%)	251 (52%)	
rs4290270 (Exon9+A57T)		A/A	A/T	T/T		A	T	
	METH	29 (18%)	80 (49%)	53 (33%)	0.840	138 (43%)	186 (57%)	0.777
	Control	49 (20%)	115 (47%)	79 (33%)		213 (44%)	273 (56%)	
Exon11+(C3)500(C2)		C3/C3	C3/C2	C2/C2		C3	C2	
	METH	159 (98%)	3 (2%)	0 (0%)	0.357	321 (99%)	3 (1%)	0.357
	Control	242 (100%)	1 (0%)	0 (0%)		485 (100%)	1 (0%)	
rs17110747 (Exon11+G654A)		G/G	G/A	A/A		G	A	
	METH	92 (57%)	63 (39%)	7 (4%)	0.956	247 (76%)	77 (24%)	0.888
	Control	136 (56%)	95 (39%)	12 (5%)		367 (76%)	119 (24%)	
rs41317114 (IVS11+G128C)		G/G	G/C	C/C		G	C	
	METH	119 (73%)	38 (23%)	5 (3%)	0.719	276 (85%)	48 (15%)	0.462
	Control	187 (77%)	50 (21%)	6 (2%)		424 (87%)	62 (13%)	

**Table 5:** Genotypic Distribution of the *TPH2* Gene SNPs in Clinically Subcategorized METH Subjects

SNP	Groups	Subgroup		N	Genotype			P
rs17110566 (IVS6+G152A)					G	G/A	A	
	Control			243	173	64	6	
	METH	Latency of Psychosis	<3 years	64	53	10	1	0.172
			≧3 years	67	47	18	2	0.966
		Prognosis of Psychosis	Transient (<1 month)	87	67	17	3	0.421
			Prolonged (≧1 month)	52	38	13	1	0.951
		Spontaneous Relapse	Not present	101	78	21	2	0.517
			Present	56	42	12	2	0.694
rs4760816 (IVS6+C6106T)					C	C/T	T	
	Control			243	57	121	65	
	METH	Latency of Psychosis	<3 years	64	13	35	16	0.771
			≧3 years	67	9	35	23	0.165
		Prognosis of Psychosis	Transient (<1 month)	87	15	39	33	0.125
			Prolonged (≧1 month)	52	7	34	11	0.107
		Spontaneous Relapse	Not present	101	19	51	31	0.577
			Present	56	8	30	18	0.306
rs4290270 (Exon9+A57T)					A	A/T	T	
	Control			243	49	115	79	
	METH	Latency of Psychosis	<3 years	64	8	35	21	0.338
			≧3 years	67	13	32	22	0.990
		Prognosis of Psychosis	Transient (<1 month)	87	16	37	34	0.541
			Prolonged (≧1 month)	52	6	34	12	0.058
		Spontaneous Relapse	Not present	101	17	52	32	0.712
			Present	56	10	27	19	0.923
rs17110747 (Exon11+G654A)					G	G/A	A	
	Control			243	136	95	12	
	METH	Latency of Psychosis	<3 years	64	35	28	1	0.438
			≧3 years	67	37	26	4	0.947
		Prognosis of Psychosis	Transient (<1 month)	87	52	31	4	0.827
			Prolonged (≧1 month)	52	26	25	1	0.366
		Spontaneous Relapse	Not present	101	57	41	3	0.712
			Present	56	32	21	3	0.970
rs41317114 (IVS11+G128C)					G	G/C	C	
	Control			243	187	50	6	
	METH	Latency of Psychosis	<3 years	64	49	15	0	0.411
			≧3 years	67	48	16	3	0.552
		Prognosis of Psychosis	Transient (<1 month)	87	65	19	3	0.852
			Prolonged (≧1 month)	52	38	13	1	0.767
		Spontaneous Relapse	Not present	101	77	21	3	0.966
			Present	56	38	17	1	0.282

N: Number of samples. P: Significance values between the METH subjects and the controls.
